# *Lactiplantibacillus plantarum* and *Saccharomyces cerevisiae*—Fermented Coconut Water Alleviates Dextran Sodium Sulfate-Induced Enteritis in Wenchang Chicken: A Gut Microbiota and Metabolomic Approach

**DOI:** 10.3390/ani14040575

**Published:** 2024-02-08

**Authors:** Leijie Zheng, Zhe Han, Jiachao Zhang, Jiamu Kang, Congfa Li, Qing Pang, Sixin Liu

**Affiliations:** 1 School of Food Science and Engineering, Hainan University, Haikou 570228, China; 15914094919@163.com (L.Z.);; 2 Key Laboratory of Food Nutrition and Functional Food of Hainan Province, Haikou 570228, China; 3 Key Laboratory of Tropical Agricultural Products Processing Technology of Haikou City, Haikou 570228, China

**Keywords:** probiotic-fermented coconut water, acute enteritis, gut microbiota diversity, metabolomics, Wenchang chicken, dextran sodium sulfate, postbiotics

## Abstract

**Simple Summary:**

In recent years, the worldwide prohibition on antibiotics, including in China, has led to an increased incidence of acute enteritis in poultry, resulting in significant economic losses. Probiotic-fermented products, especially postbiotics, have exhibited promising probiotic effects, demonstrating potential for ameliorating acute enteritis. It has been reported that probiotic and postbiotics intervention can effectively treat acute enteritis. In reality, coconut water, an abundant local resource in Hainan, China, is frequently easy to be contaminated and consequently underutilized in industrial processes. In this study, Wenchang chicken was employed as an DSS-induced model of acute enteritis and then fed with coconut water fermented by probiotics. The results show that coconut water fermented by probiotics facilitates the recovery of production performance in afflicted chickens, improving serum immune and biochemical indexes, intestinal tissue structure, and the composition of the gut microbiota structure and metabolomics. This study provides theoretical insights for the development and utilization of new fermented feed resources known as postbiotics and provides a reference for the sustainable green development of the poultry breeding industry.

**Abstract:**

In order to investigate the potential mechanisms of probiotic-fermented coconut water in treating enteritis, this study conducted a comprehensive analysis of the effects of probiotic intervention on the recovery from Dextran Sodium Sulfate-induced acute enteritis in Wenchang chicks. The analysis encompassed the assessment of growth performance, serum indicators, intestinal tissue structure, and metagenomic and metabolomic profiles of cecal contents in 60 Wenchang chicks subjected to intervention. This approach aimed to elucidate the impact of probiotic intervention on the recovery process from acute enteritis at both the genetic and metabolic levels in the avian model. The results revealed that intervention with *Saccharomyces cerevisiae* Y301 improved the growth rate of chicks. and intervention with *Lactiplantibacillus plantarum* MS2c regulated the glycerophospholipid metabolism pathway and reshaped the gut microbiota structure in modeling chicks with acute enteritis, reducing the abundance of potentially pathogenic bacteria from the *Alistipes* and increasing the abundance of potentially beneficial species from the Christensenellaceae. This intervention resulted in the production of specific gut metabolites, including Gentamicin C and polymyxin B2, recognized for their therapeutic effects on acute enteritis. The combined intervention of *S. cerevisiae* Y301 and *L. plantarum* MS2c not only enhanced growth performance but also mitigated intestinal wall damage and increased the abundance of gut metabolites such as gentamicin C and polymyxin B2, thereby mitigating symptoms of enteritis. Furthermore, this combined intervention reduced the levels of serum immune markers, including IL-10, IL-6, TNF-α, IFN-γ, and D-lactic acid, thus mitigating intestinal epithelial cell damage and promoting acute enteritis recovery. This study provides crucial insights into the mechanisms of action of probiotics and probiotic-fermented coconut water in acute enteritis recovery, offering new perspectives for sustainable farming practices for Wenchang chicken.

## 1. Introduction

Gastrointestinal diseases, such as acute enteritis in animals, are often characterized by various intestinal inflammations and physiological and biochemical abnormalities [1]. These abnormalities manifest as reduced food intake, impaired weight gain, dysregulation of the immune system, and elevated levels of inflammatory factors in the serum [2]. These physiological and immune responses result in intricate biochemical changes within the organism. Furthermore, acute enteritis disrupts the balance of the intestinal microbiota, thereby increasing the susceptibility to pathogenic microbial infections and adversely affecting animal health [3,4].

Wenchang chicken, a distinct poultry breed in Hainan Province, China, is highly sought after for its unique flavor and high-quality meat. However, the Wenchang chicken industry faces significant health challenges with the prohibition of antibiotics. Acute enteritis is a prevalent intestinal disease in the broiler chicken sector of the poultry farming industry [5,6]. This disease damages the intestines of chickens, affecting food digestion, absorption, and the normal functioning of the immune system [7]. The prolonged growth period of Wenchang chickens exposes them to an increased susceptibility to acute enteritis [8]. Consequently, there is an urgent need to develop new farming techniques that reduce acute enteritis occurrence and promote self-recovery. This approach not only aids in reducing production costs but also paves the way for establishing a sustainable and healthy farming model.

Probiotics, extensively studied for their efficacy in treating acute enteritis, are renowned for their ability to maintain gut microbiota balance, boost immune system activity, improve intestinal mucosal barrier function, and alleviate inflammation. In the treatment of acute enteritis, probiotics have been widely investigated, demonstrating capabilities such as maintaining gut microbiota balance, enhancing immune system activity, improving intestinal mucosal barrier function, and reducing inflammation [9,10,11]. However, it is important to note that different strains of probiotics have varying effects. Therefore, the careful selection of optimal probiotic strains and the proper utilization of their metabolic activities are crucial in harnessing their benefits effectively [12]. Probiotic products are typically available in the form of oral supplements, and they can also be incorporated into feed or drinking water in the poultry industry, depending on the specific strain and product characteristics.

The Hainan province of China, as well as several Southeast Asian countries, are renowned for their coconut production. During coconut kernel processing, coconut water is subsequently generated, which is often considered a byproduct that is inconvenient to preserve and unsuitable for consumption. In this research, the investigation focuses on the use of coconut water as a medium for probiotic cultivation, leading to the production of prebiotic products. In exploring the beneficial role of probiotics in promoting the recovery of acute enteritis in Wenchang chicks, this study utilized dextran sodium sulfate (DSS) modeling as a method to induce acute enteritis in Wenchang chicks [13,14,15,16].This study further investigated the efficacy of these probiotic products in facilitating the recovery from acute enteritis in the Wenchang chick model. This research not only sheds light on the impact and mechanisms of probiotic fermentation in coconut water on the self-recovery of chicks with acute enteritis but also presents a valuable pathway for the utilization of non-fresh, inedible coconut water. Moreover, it explores novel approaches for the sustainable farming of Wenchang chicks.

## 2. Materials and Methods

### 2.1. Strains and Fermentation Conditions

*Lactiplantibacillus plantarum* MS2c, a strain exhibiting potent probiotic activity, was isolated from the gut microbiota of Wenchang chicks. *Saccharomyces cerevisiae* Y301 was isolated from naturally fermented coconut water. The fermentation conditions involved a 3% (*v*/*v*) inoculation of MS2c at 37 °C for 24 h; Y301 was fermented at 30 °C; and the mixed group was fermented at 34 °C with an equal fermentation ratio of 1:1 for both strains. The viable cell count was 2 × 10^9^ CFU/mL for MS2c-fermented coconut water and 5 × 10^8^ CFU/mL for Y301-fermented coconut water.

### 2.2. Animals and their Management

Sixty Wenchang chicks, comprising 2-week-old roosters with a weight range of 90–110 g, were procured in Wenchang, Hainan. These chicks were randomly divided into six groups (n = 10): the blank control group (CK1) was fed a normal diet and provided pure water; the model group (CK2) had unrestricted access to a 1.5% dextran sulfate sodium solution (*w*/*v*, 40,000 kDa, Macklin) for 7 days and then orally received the corresponding solution for two weeks; the fresh coconut water group (CK3) was fed normal diet along with fresh coconut water; the Y3 group received *S.cerevisiae* Y301 fermented coconut water in addition to their normal diet; the M2 group was given coconut water fermented by *L. plantarum* MS2c on top of their normal diet; the mixed group (My5) was provided coconut water fermented by both *L. plantarum* and *S.cerevisiae* on their normal diet (Table 1). All chicks freely consume non-antibiotic commercial feed. Except for the control and model groups, the other four groups had unrestricted exposure to a 1.5% dextran sulfate sodium solution [15] (*w*/*v*, 40,000 kDa, Macklin) for 7 days before the start of the experiment for modeling purposes, followed by simultaneous initiation of the experiment and receiving their respective interventions for two weeks after the modeling period. On the 21st day, a 24 h fasting period was observed, followed by weighing and blood collection from the wing vein. Euthanasia was performed using cervical bleeding, and specimens were collected. The animal experiment and experimental protocol were approved by the Ethics Committee of Hainan University, China, and followed the guidelines of the “Laboratory Animals Protection and Utilization Guidelines of Hainan University” (Approval No. HNUAUCC-2023-00210).

### 2.3. Physiological Indicators

Physiological indicators were used to assess the chicks’ health, including daily feed intake, daily weight gain, and the Disease Activity Index (DAI), as described in Table 2. Fecal potential blood levels were determined using the kit. Before the chicks were euthanized, blood samples were collected from ten chicks in each group. Blood was allowed to clot naturally for 30 min before the separation of serum. Serum levels of the inflammatory cytokines IL-10, IL-6, TNF-α, and IFN-γ were determined using ELISA kits (Solabo, Beijing, China). Serum chemical parameters, including GOT (Glutamic Oxaloacetic Transaminase), GPT (Glutamic Pyruvic Transaminase), UA (Uric Acid), and D-lactic acid, were also measured using assay kits (Solabo, Beijing, China).

### 2.4. Pathological Indicators

After the experiment, a 1 cm segment of the cecum at the terminal end of the ileum was harvested. This segment was used to assess the cecal wall thickness and was subjected to H&E staining. Following the protocol described by the method of O. Srinual for staining chick intestinal tissue [17], the cecal tissue was fixed in 4% paraformaldehyde (*w*/*v*) for 24 h. Subsequently, it was dehydrated, embedded in paraffin, and cut into 3 μm sections. Some colon samples were stained with hematoxylin and eosin (H&E) as described earlier [18,19,20,21]. Tissue pathology was assessed using optical microscopy (Nikon Corporation, Japan), and histological injury scores were calculated to evaluate the extent of tissue damage and analyzed using Image-Pro Plus 6.0 analysis software, with results reported in millimeters.

### 2.5. Extraction and Diversity Analysis of the Cecal Content Microbiota

After the chicks were euthanized, the cecal content was quickly extracted from six randomly selected chicks in each group. The cecal content samples were preserved in dry ice and sent to Shanghai Meiji Biological Co., Ltd. (Shanghai, China), for cecal microbiota diversity analysis. The analysis involved DNA extraction, PCR amplification of the V3-V4 variable region of the 16S rRNA gene, library construction, and high-throughput sequencing for 16S functional prediction analysis.

The upstream primers used were 338F (5′-ACTCCTACGGGAGGCAGCAG-3′), and the downstream primers used were 806R (5′-GGACTACHVGGGTWTCTAAT-3′). The 16S rRNA gene V3-V4 variable region of the samples was amplified by PCR, and the PCR reaction system was as follows: 5× TransStart FastPfu Buffer 4 μL, 2.5 mM dNTPs 2 μL, upstream primers (5 μM) 0.8 μL, downstream primers (5 μM) 0.8 μL, TransStart FastPfu DNA Polymerase 0.4 μL, template DNA 10 ng, adjusted to 20 μL. Three replicates were prepared for each sample.

### 2.6. Cecal Content Metabolomics Analysis

Cecal content samples were placed in 2 mL Eppendorf tubes and stored at −80 °C. The samples were sent to Shanghai Meiji Biological Co., Ltd., for untargeted LC-MS metabolomics profiling [22]. Sample preparation involved grinding the samples for liquid sample extraction. Quality control samples were prepared by mixing an equal volume of all the metabolites to assess the repeatability of the analysis process. The analysis was conducted using a mass spectrometer.

For the LC separation, 2 μL of each sample was injected into the HSS T3 column (100 mm × 2.1 mm i.d., 1.8 µm), with the flow rate set to 0.4 mL/min and the column temperature maintained at 40 °C. The mobile phase A consisted of 95% water and 5% acetonitrile (containing 0.1% formic acid), and the mobile phase B consisted of 47.5% acetonitrile, 47.5% isopropanol, and 5% water (containing 0.1% formic acid).

The mass spectrometry signal acquisition for the samples was conducted in positive and negative ion scan modes, with a mass scan range of 70–1050 *m*/*z*. The instrument parameters included a sheath gas flow rate of 50 psi, an auxiliary gas flow rate of 13 psi, an auxiliary gas heater temperature of 425 °C, a positive mode ion spray voltage of 3500 V, a negative mode ion spray voltage of −3500 V, and a collision energy in the range of 20–40–60 V in the dynamic multiple reaction monitoring (dMRM) mode. Data were collected in data-dependent acquisition (DDA) mode, with the first-order mass spectrometry resolution set at 60,000 and the second-order resolution at 7500.

### 2.7. Bioinformatics and Statistical Analysis

GraphPad (GraphPad Software 9, San Diego, CA, USA) and R software (R 4.3.1, R studio 1.3.959) were used for data analyses. Data were presented as mean ± standard deviation (SD), and comparisons were carried out using the Wilcoxon signed-rank test, with significant differences denoted by symbols. Differences between lowercase letters were considered as *p* < 0.05, and significance levels were indicated as follows: *: *p* < 0.05, **: *p* < 0.01, and ***: *p* < 0.001, representing significant, very significant, and highly significant differences, respectively.

## 3. Results

To comprehensively study the recovery effect of two probiotics on acute enteritis, acute enteritis was induced in chicks in this study. First, a modeling experiment involving ad libitum drinking was conducted in 14-day-old chicks to assess the effects of two different concentrations (1.5% and 2.5% *w*/*v*) and two intake methods (gavage and free drinking) of sodium dextran sulfate (DSS) on chicks. The results showed that 14-day-old chicks, which freely drank a 1.5% DSS solution for 7 days, exhibited symptoms including bloody stools and diarrhea. However, the higher concentration of 2.5% DSS solution caused more severe symptoms and even resulted in death. Hence, the 1.5% DSS concentration was chosen for free drinking over a period of 7 days to induce the acute enteritis model.

**Figure 1:**
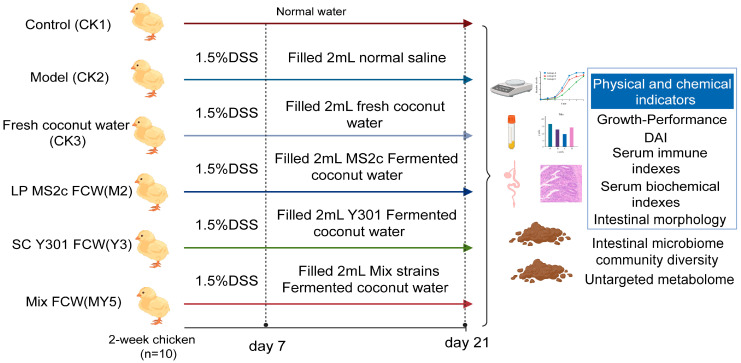
Intervention experiment design: coconut water fermented by probiotics.

Next, 60 14-day-old chicks were randomly divided into six groups, each consisting of 10 chicks. All six groups conduct the experiment simultaneously after the 7-day modeling period and received their respective interventions for a duration of two weeks (Figure 1).

### 3.1. Effect of Probiotic Intervention on Growth Performance Recovery in the Chick Acute Enteritis Model

During the initial 7 days of DSS ingestion, the groups CK2, CK3, M2, Y3, and MY5 showed lower feed intake compared to the control group. However, by the 12th day, the MY5 and Y3 groups displayed a faster recovery in feed intake compared to the CK2, CK3, and M2 groups. On the 21st day, the MY5 and Y3 groups exhibited higher feed intake than the M2, CK2, and CK3 groups. The average body weight and feed intake of the chicks exhibited a correlated trend. After 21 days of intervention, compared to the model group, the average body weight of chicks in the Y3 and MY5 groups receiving Y301 fermented coconut water was higher than that of the MS2c group.

**Figure 2:**
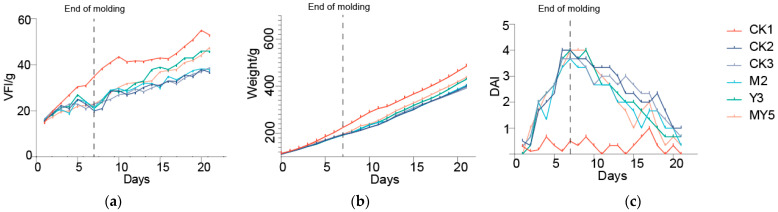
Effect of probiotic-fermented coconut water on growth performance of chicks. (**a**) Average daily feed intake. (**b**) Average daily weight (**c**) Average DAI index.

Both feed intake and body weight of the chicks in the probiotic intervention groups showed a recovery trend, increasing more quickly than the model group (Figure 2a,b). Additionally, the intervention with probiotic fermentation in coconut water improved the Disease Activity Index (DAI), specifically the score related to intestinal diseases. The experimental results revealed that the probiotic intervention effectively improved the abnormal feces, with the groups that received probiotic intervention showing a significantly faster recovery compared to the CK2 group (Figure 2c).

### 3.2. Impact of Probiotic Intervention on Physiological Indicators in the Chick Acute Enteritis Model

After exploring the impact of different probiotic-fermented coconut waters on the growth performance of chicks with acute enteritis, this study shifted its focus to immune indicators. To assess the influence of coconut water supplemented with probiotics (M2, Y3, and MY5 groups) on serum immune function, the levels of various immune factors and biochemical indicators were analyzed in the serum of 10 experimental chicks from each group.

**Figure 3:**
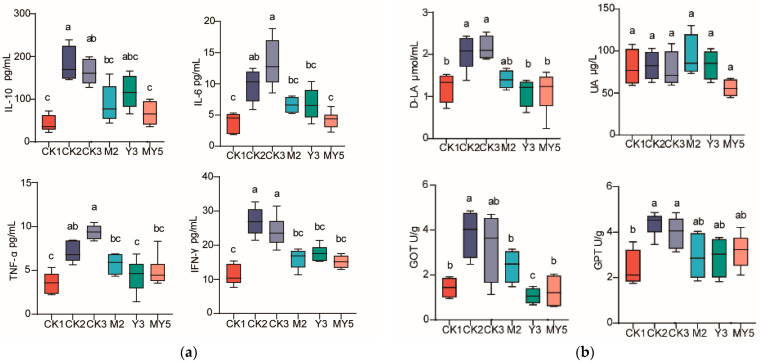
Effect of fermented coconut water on serum immune biochemical indexes. (**a**) Serum immune index content map; (**b**) Serum biochemical index content map. Letters: Groups that do not share the same letter are significantly (*p* < 0.05) different from each other.

To evaluate the impact of probiotic fermentation in coconut water on serum immune factors, a range of immunological and biochemical indicators were measured in the serum of each group, each containing 10 experimental chicks. This intervention resulted in reduced levels of both inflammatory and anti-inflammatory factors. Regarding the IL-10 indicator, the serum IL-10 levels in the M2 and MY5 groups were significantly lower than in the CK2 group (*p* < 0.05). For the IL-6 indicator, the MY5 group exhibited significantly lower levels compared to the CK2 group (*p* < 0.05). In terms of the TNF-α indicator, the Y3 group displayed significantly lower levels than the CK2 group (*p* < 0.05). As for the IFN-γ indicator, the levels in the M2, Y3, and MY5 groups were significantly lower than in the CK2 group (*p* < 0.05). The levels of immune factors in the serum are closely associated with the inflammatory response of the chick body (Figure 3a). The level of serum D-lactic acid can be used as a characteristic index of chick intestinal leakage. The experimental results indicated that the D-lactic acid levels in the serum of the CK2 group were significantly higher compared to the probiotic intervention groups Y3 and MY5 (*p* < 0.05). The intervention of Y301-fermented coconut water helped alleviate the levels of D-lactic acid in the serum. The levels of uric acid (UA) in the serum can reflect the kidney load. Probiotic fermentation in coconut water did not significantly affect serum uric acid levels in chicks, with no significant intergroup differences observed (*p* > 0.05). This lack of effect may be attributed to individual variations in chicks. Probiotic fermentation in coconut water (M2, Y3, and MY5) significantly reduced GOT levels (*p* < 0.05) and suppressed GPT elevation in experimental chicks. D-lactic acid paralleled the trends observed for IL-10 and IFN-γ, suggesting the feasibility of establishing an intestinal inflammation model (Figure 3b).

### 3.3. Effects of Probiotic Intervention on the Repair of Intestinal Wall Damage in the Chick Acute Enteritis Model

**Figure 4:**
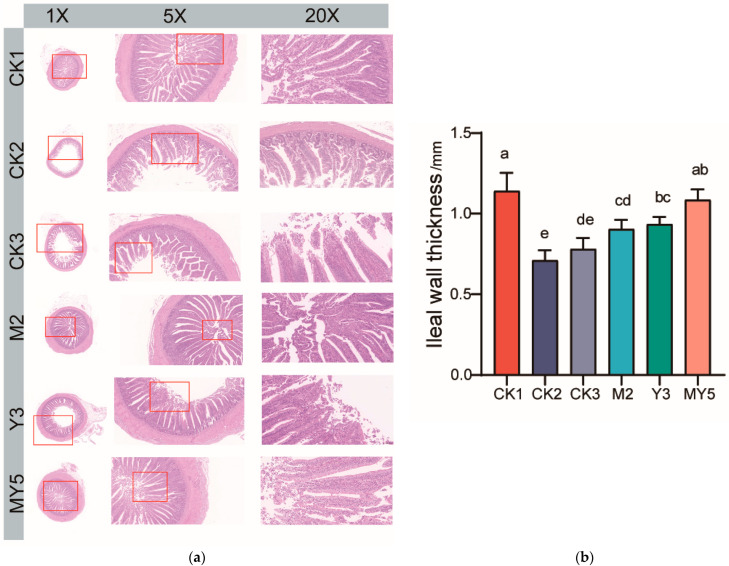
Effects of Probiotic Fermentation in Coconut Water Intervention on Repair of Intestinal Wall Damage in Chick Acute Enteritis Model (**a**) Effect of Probiotic-Fermented Coconut Water on Intestinal Histomorphology of Chick Acute Enteritis Model (**b**) Comparison of Intestinal Wall Thickness. Letters: Groups that do not share the same letter are significantly (*p* < 0.05) different from each other.

In the histological examination of the ileum by HE staining (Figure 4a), it was observed that DSS induction caused substantial loss of ileum epithelial cells, disruption of villous structures, and extensive villous cell fragmentation.

According to the results presented in Figure 4b, it is evident that there was no significant difference in intestinal wall thickness between the MY5 group and the control group (*p* > 0.05). This suggests that the combined intervention of the specific MS2c and Y301 fermentations in coconut water significantly promoted the self-recovery of chicks with acute enteritis. After three weeks of intervention, intestinal wall thickness exhibited significant improvement toward normal levels, although complete restoration was not yet achieved. The Y3 group and the M2 group showed significantly greater increases in intestinal wall thickness compared to the model group (CK2); however, it remained significantly lower than that of the control group (CK1).

### 3.4. Effects of Probiotic Intervention on Gut Microbial Diversity in the Chick Acute Enteritis Model

**Figure 5:**
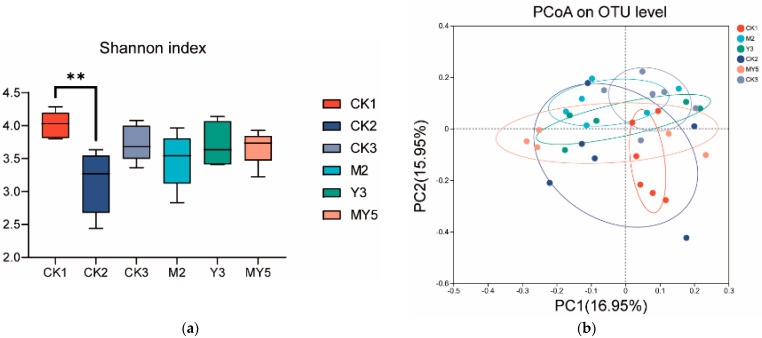
Shannon index-α diversity (**a**) and β diversity (**b**) of intestinal metagenome-α diversity. ** indicates significant difference (*p* < 0.01).

The metagenome of the cecum contents of experimental chicks was determined, and the α diversity and β diversity were analyzed (Figure 5a,b), which revealed the only significant difference in α diversity observed between the CK1 group and the CK2 group (*p* < 0.01). This indicated that the DSS intervention (CK2 group) significantly reduced the community diversity of cecal content in chicks.

**Figure 6:**
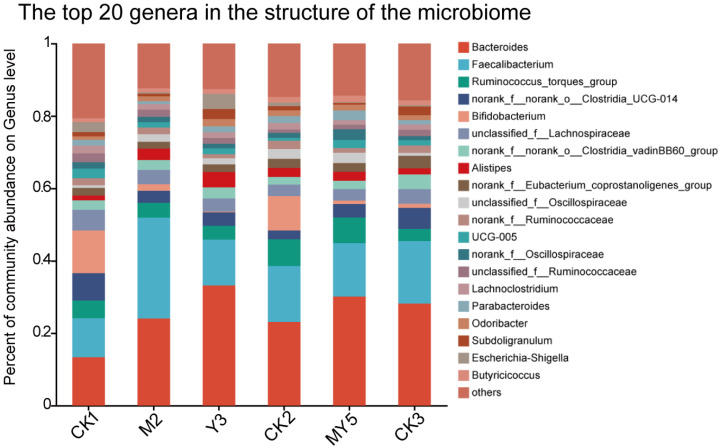
Structure diagram of cecal contents microbiota.

Upon analyzing the microbial composition (Figure 6), it was observed that *Bifidobacterium*, Lachnospiraceae, *Alistipes*, and Escherichia-Shigella play important roles in the gut microbial structure. Escherichia-Shigella, a common pathogen causing bacterial dysentery, exhibited higher abundance in the CK1 (2.7%) and Y3 (3.6%) groups compared to the M2 and MY5 groups (0.3% and 0.16%).

**Figure 7:**
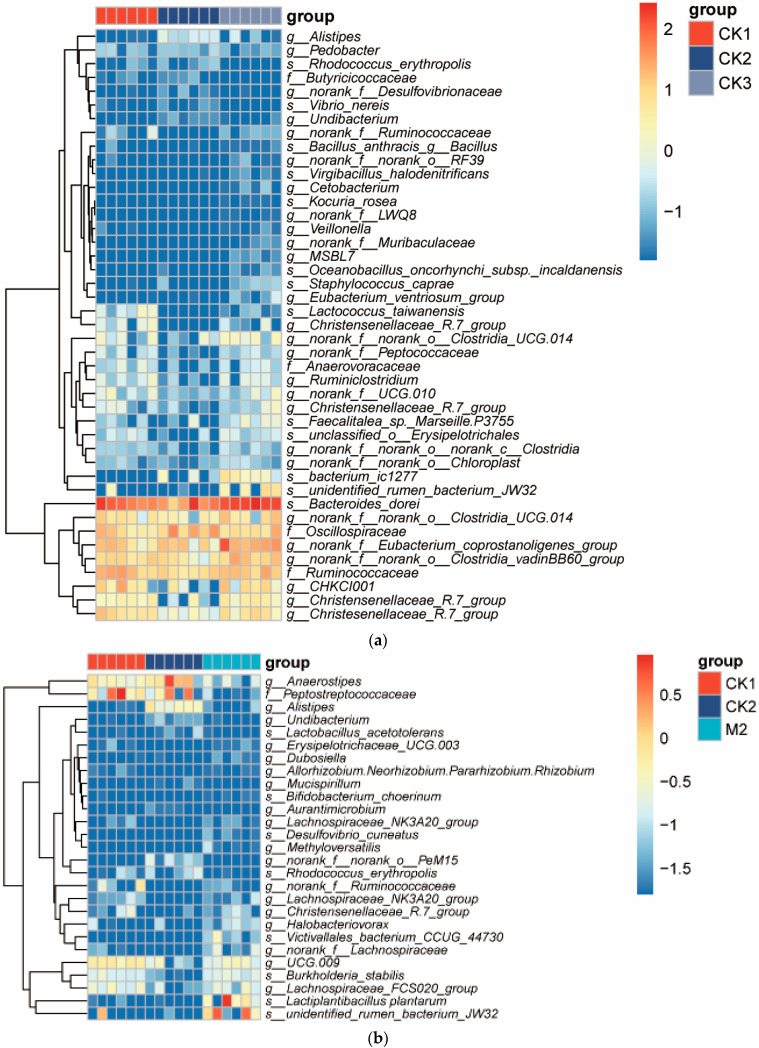

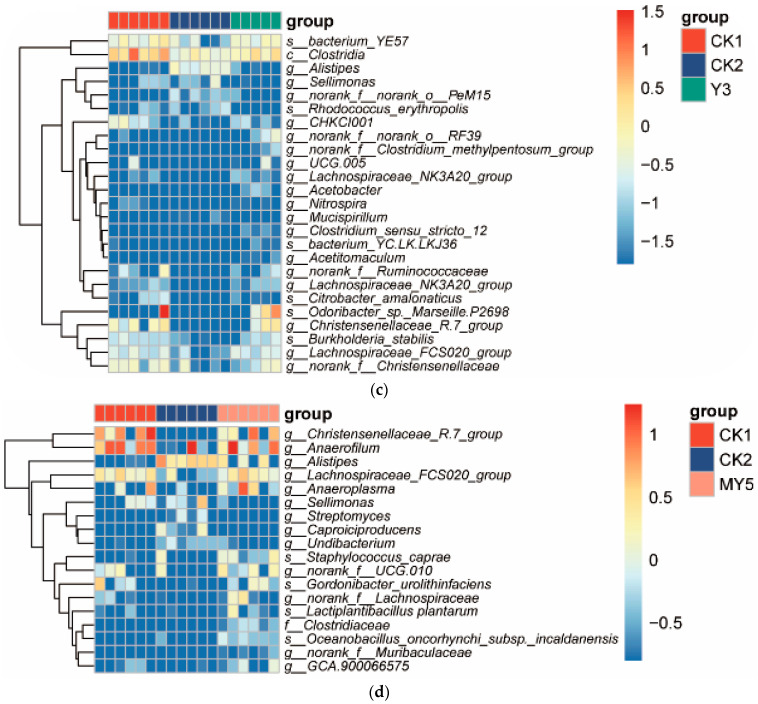
The effect of fermented coconut water on microbial community in cecum contents was explained. (**a**) CK1 vs. CK2 vs. CK3; (**b**)CK1 vs. CK2 vs. M2; (**c**) CK1 vs. CK2 vs. Y3; (**d**) CK1 vs. CK2 vs. MY5.

In the group analysis (Figure 7a), genera that showed significant differences (*p* < 0.05) were selected for further analysis. Among the CK1, CK2, and CK3 groups, the abundance of *Bacteroides dorei* was higher, which did not undergo MS2c and Y301 interventions. In the CK2 group, the abundance of *B. dorei* was lower. Another important gut genus is *Alistipes*. In the CK2 group, its abundance was higher, while it was lower in the control groups, CK1 and in the fresh coconut water CK3 groups (*p* < 0.05). *Lactococcus taiwanensis* was significantly more abundant in the control groups (CK1 and CK2) than in the other groups (*p* < 0.01). Christensenellaceae belongs to the Firmicutes phylum, and its abundance was higher in the control groups. However, DSS treatment dramatically reduced its abundance.

For the M2 group, the heatmap (Figure 7b) shows that the abundance of *Alistipes* was lower. MS2c intervention can reduce the abundance of Peptostreptococcaceae in the chick cecum. Moreover, *L. plantarum* had a higher proportion in the M2 group compared to the CK1 and CK2 groups.

The heatmap (Figure 7c) reveals relatively higher abundances of Clostridia in the Y301 group. The Firmicutes phylum had lower abundances in the Y301 intervention group and higher abundances in the model group, indicating that the Y301 intervention significantly affected the abundance of Firmicutes.

For the MY5 group, the Christensenella ceae exhibited higher relative abundance in both the control group and the composite strain intervention group (Figure 7d). This suggests greater stability of the gut microbiota in the CK1 and MY5 groups. Lachnospiraceae, a family known for its production of short-chain fatty acids and negative association with acute enteritis, exhibited higher abundances in the CK1 and MY5 groups compared to the CK2 group. This suggests that the gut microbiota in the MY5 group is more similar to a healthy gut microbiota. *L. plantarum* also had a higher abundance in the MY5 group, indicating that the intervention with the composite strains-fermented coconut water is beneficial for gut microbiota stability.

### 3.5. Effects of Probiotic Intervention on the Metabolites in the Cecal Content of the Chick Acute Enteritis Model

**Figure 8:**
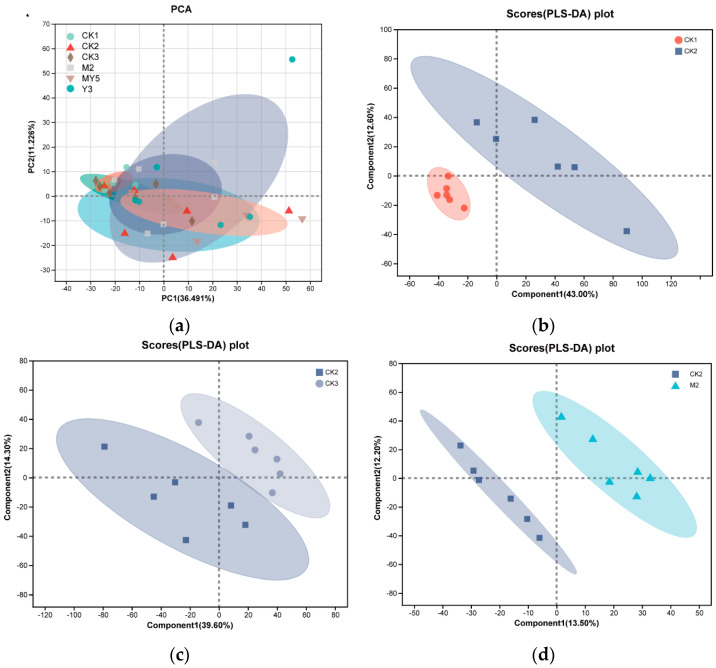

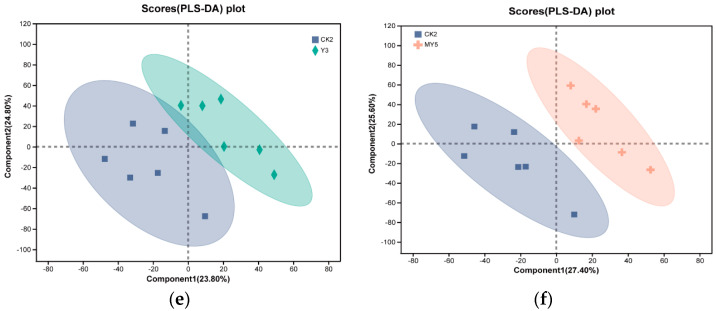
PCA diagram of six groups and PLS-DA diagram for comparisons between each group and the model group. (**a**) PCA diagram between six groups: (**b**) CK1 and CK2; (**c**) CK2 and CK3; (**d**) CK2 and M2; (**e**) CK2 and Y3; (**f**) CK2 and MY5.

In the comparative analysis of the metabolites, a principal component analysis (PCA) were performed based on the combined data of anions and cations from the CK1, CK2, CK3, M2, Y3, and MY5 groups (Figure 8). The aim of the PCA was to reduce the dimensionality of the data. The results showed that the metabolites of the different groups were closely clustered together, indicating a high degree of similarity among the models. However, performing partial least squares-discriminant analysis (PLS-DA) on the experimental groups revealed significantly greater discrimination between the CK1, M2, and MY5 groups compared to CK2 (*p* < 0.05), suggesting a notable impact of the interventions. Moreover, the separations of the CK3 and Y3 groups from the CK2 group were relatively lower. This suggests that the limited influence of CK3 and Y3 water on gut metabolites may be due to probiotic fermentation with MS2c, which was likely the primary driver of differences in gut metabolic products.

In the Variable Importance in Projection (VIP) analysis, metabolites with notable variances among the groups were pinpointed. Through cross-referencing various databases, the specific compounds of these metabolites were precisely identified. Among these metabolites, the analysis focused on those showing extremely significant differences (*p* < 0.01).

**Figure 9:**
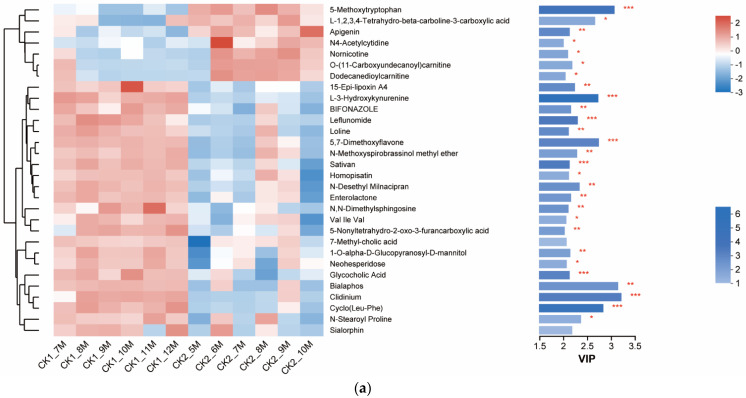

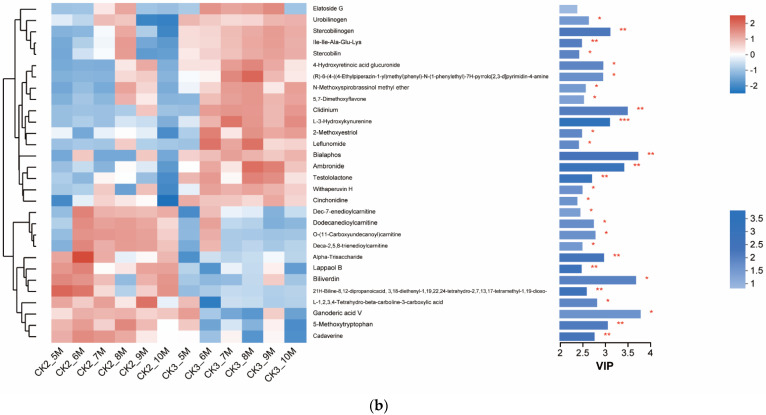
Differential metabolites between control groups (**a**) Comparison between CK1 and CK2 (**b**) Comparison between CK2 and CK3. * *p* < 0.05; ** *p* < 0.01; *** *p* < 0.001.

In the VIP plot of the CK1 group versus the CK2 group (Figure 9a), 19 metabolites were observed, including 5-methoxytryptophan, apigenin, 15-epi-lipoxin A4, L-3-hydroxykynurenine, and so on, which exhibited highly significant differences. When comparing the differences between the CK1 and CK2 groups, it was noted that the CK2 group had fewer significantly abundant differential metabolites, whereas the CK1 group had more.

In the CK3 group (Figure 9b), the abundance of 7 metabolites, such as stercobilinogen and clidinium, was significantly higher than in the CK2 group. Most of these metabolites are products of normal metabolic pathways in the chick, such as fatty acids.

**Figure 10:**
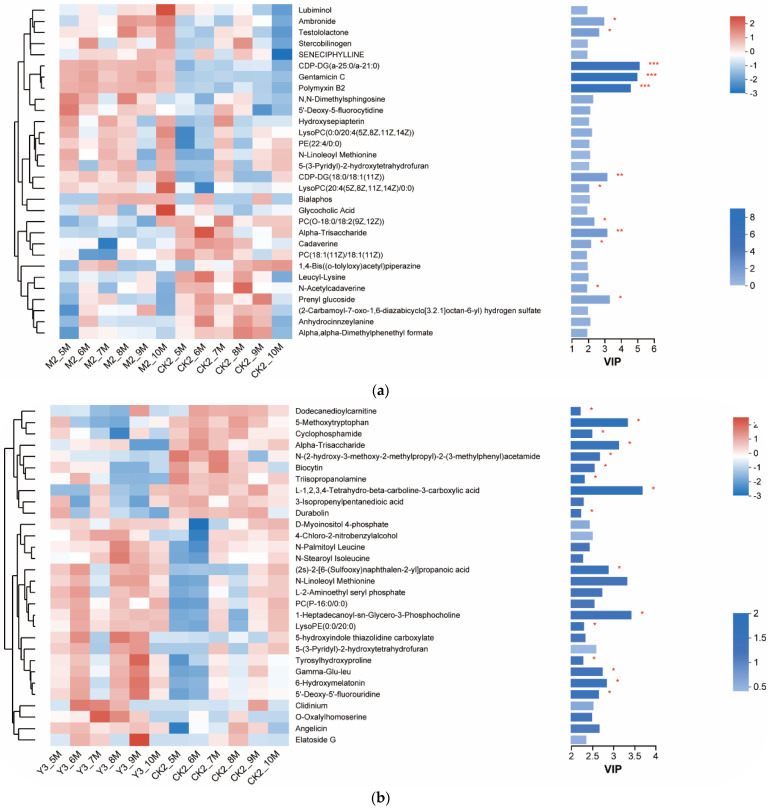

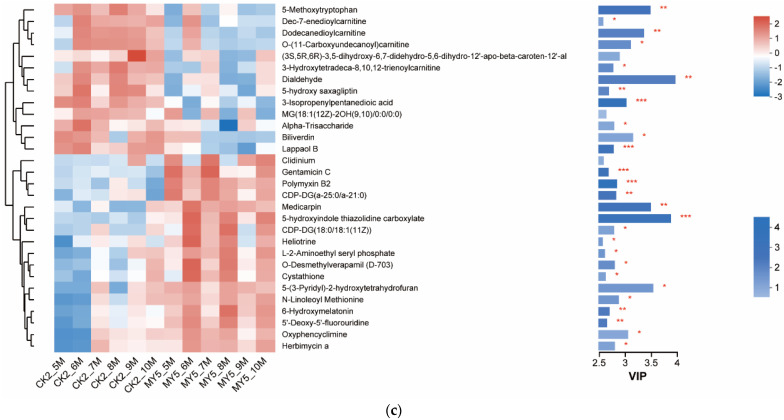
Differential metabolites between control groups (**a**) Comparison between CK2 and M2 (**b**) Comparison between CK2 and Y3 (**c**) Comparison between CK2 and MY5. * *p* < 0.05; ** *p* < 0.01; *** *p* < 0.001.

As shown in Figure 10a, it was observed that there were extremely significant differences in metabolites (Gentamicin C, Polymyxin B2, CDP-DG (a-25:0/a-21:0), CDP-DG (18:0/18:1 (11Z), and Alpha-Trisaccharide) between the M2 group and the CK2 group. In the M2 group, these metabolites were significantly more abundant compared to the model group (CK2). Notably, Gentamicin C and Polymyxin B2 are normally drugs currently used to treat acute enteritis and intestinal cancer. CDP-DG is a type of glycerophospholipid related to cell membranes and active substances on cell membranes.

In the Y3 group (Figure 10b), while there were no extremely significant differences in the metabolites abovementioned, there were significant differences (*p* < 0.05) in some other metabolites, including dodecanedioylcarnitine, 5-methoxytryptophan, cyclophosphamide, 6-hydroxymelatonin, and 5′-deoxy-5′-fluorouridine.

In the MY5 group (Figure 10c), it shows extremely significant higher abundances in metabolites including Gentamicin C, Polymyxin B2, CDP-DG (a-25:0/a-21:0), medicarpin, 5-hydroxysaxagliptin, 6-hydroxymelatonin, 5′-deoxy-5′-fluorouridine, 3-isopropenylpentanedioic acid, 5-methoxytryptophan, dodecanedioylcarnitine, and dialdehyde as compared with the model group.

### 3.6. Metabolic Pathway Analysis of Probiotic Intervention on Intestinal Inflammation in Model Chicks

**Figure 11:**
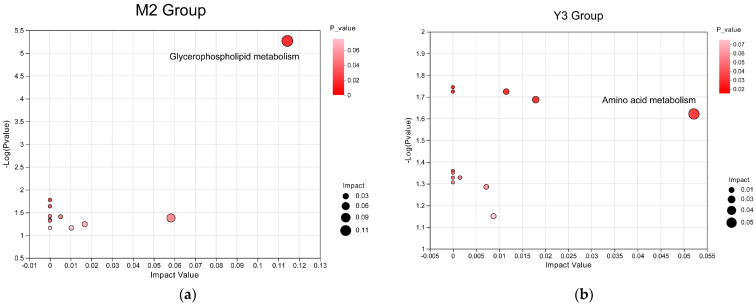
Displays the KEGG pathway topological maps. (**a**) is the KEGG pathway topological map for the M2 group, and (**b**) is the KEGG pathway topological map for the Y3 group.

In the topological map, the horizontal axis represents importance, and the vertical axis represents *p*-values. Metabolic pathways with *p*-values less than 0.05 were selected, with those demonstrating the greatest significance identified as key pathways influencing metabolite activity. In the case of MS2c intervention, the most crucial metabolic pathway is glycerophospholipid metabolism (Figure 11a). Conversely, in the case of the Y301 intervention, the primary pathways affecting metabolites include the alanine, aspartate, and glutamate metabolism pathways (Figure 11b).

### 3.7. Correlation Analysis between Differential Microbiota and Differential Metabolites

The relationship between differential microbiota and metabolites was further investigated based on *p*-values under 0.05 and correlation coefficients (r) exceeding 0.4. These thresholds were chosen to identify robust and statistically significant associations between differential microbiota and differential metabolites for further in-depth exploration of their interactions.

**Figure 12:**
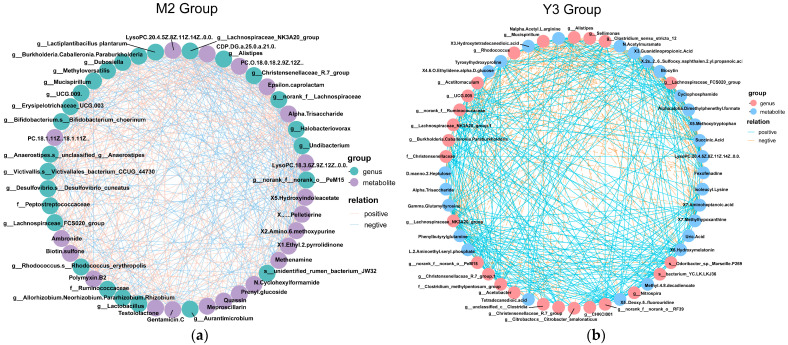
Depicts the network analysis of differential microbiota and differential metabolites. (**a**) shows the correlation analysis of differential microbiota and differential metabolites in the case of single-strain MS2c fermentation of coconut water for intervention in chick colitis models. (**b**) represents the correlation analysis of differential microbiota and differential metabolites when single-strain Y301 ferments coconut water for intervention in chick colitis models.

The analysis of associations with differential metabolites (Figure 12a) revealed a significant negative correlation between MS2c intervention and the genus *Alistipes*. This finding indicates that MS2c-fermented coconut water can reduce the abundance of *Alistipes* in the cecum, further supporting its inhibitory effect on pathogenic bacteria in the gut.

In the gut contents, several substances were found to exhibit a positive correlation with MS2c-fermented coconut water, including the Christensenellaceae family, gentamicin C, CDP-DG (a-25:0/a-21:0), Lachnospiraceae family, polymyxin B2, ambronide, and α-Trisaccharide.

In the correlation analysis of the Y3 group (refer to Figure 12b), a positive correlation was observed between 5-methoxytryptamine and the genus *Alistipes*. Additionally, an enrichment of both the Christensenellaceae and Lachnospiraceae families was noted in the Y3 group, which showed positive correlations with X7-Aminoheptanoic acid, X6-Hydroxymelatonin, and X5-Methoxytryptamine.

### 3.8. Promotion Mechanisms of Probiotic Intervention on the Recovery of Acute Enteritis Model Chicks

**Figure 13:**
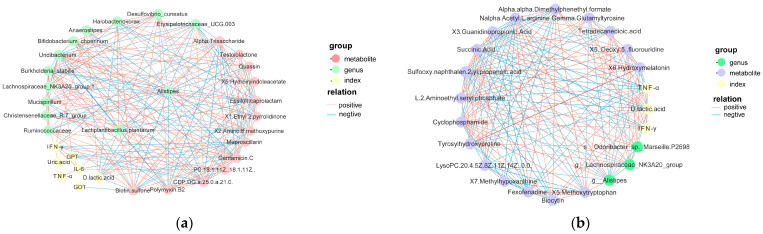
Illustrates the mechanisms by which MS2c and Y301 promote recovery in chick colitis models. (**a**) Outlines the mechanism of MS2c, while (**b**) Depicts the mechanism of Y301.

In the correlation analysis of serum immune indicators, differential metabolites, and distinct microbial communities, it was observed that in the M2 group (Figure 13a), there is a positive correlation between *L. plantarum* and Christensenellaceae, while *Alistipes* shows a negative correlation with *L. plantarum*. Additionally, *L. plantarum* exhibits a positive correlation with CDP-DG (a-25:0/a-21:0), gentamicin C, and polymyxin B2. Furthermore, serum indicators such as IFN-γ, IL-6, and TNF-α are directly or indirectly positively correlated with *Alistipes*. In the Y3 group (Figure 13b), the increased abundance of Lachnospiraceae is negatively correlated with *Alistipes*, and it is positively correlated with Hydroxymelatonin and fluorouridine abundance. Moreover, it is negatively correlated with the abundance of D-lactic acid.

## 4. Discussion

The findings of this study suggest that feeding chicks with probiotic-fermented coconut water may be beneficial in promoting their recovery from acute enteritis. It was observed that probiotic-fermented coconut water as an intervention improved the physical, chemical, and biological barriers of the intestine, thereby facilitating the recovery from acute enteritis in Wenchang chicks with DSS-induced acute enteritis.

After freely consuming a 1.5% DSS solution for 7 days, chicks in the model group showed significantly increased levels of the immune factors IL-10, IL-6, TNF-α, and IFN-γ. This elevation contributed to increased D-lactic acid levels and GOT levels in the liver, playing a role in liver damage [23]. This imbalance in the diversity of intestinal microbial communities contributed to an elevated Disease Activity Index (DAI) in the chicks [24]. The observed effects included a decrease in average daily feed intake, reduced daily weight gain, a lethargic state, and the presence of bloody and loose stools. These observations are consistent with the description of an acute enteritis model chick in the literature [15].

Histological examination of intestinal tissue slices revealed that DSS disrupted the epithelial tissue of the intestinal wall, causing cell rupture and loss of cellular function. The destruction of intestinal epithelial cells impaired the chicks’ utilization and digestive functions of the feed, leading to decreased weight gain and reduced digestive efficiency.

According to the literature, the expression of pro-inflammatory cytokines leads to intestinal and mucosal inflammation [25]. Therefore, reducing pro-inflammatory cytokines is considered an effective approach for treating acute enteritis [26,27]. This study reinforces the observation that probiotic intervention inhibits the levels of anti-inflammatory cytokines. Notably, a decrease in pro-inflammatory cytokines like IL-6, TNF-α, and IFN-γ was observed after the administration of a 1.5% DSS solution.

In summary, it was observed that the levels of immune factors were higher in the CK2 and CK3 groups, supporting the validity of the model. Additionally, it was found that fresh coconut water alone did not significantly contribute to the reduction of inflammatory factors. Taking into account the average body weight and average feed intake of the chicks, it can be speculated that the intestinal inflammation was potentially more severe, leading to decreased appetite and lethargy, ultimately negatively impacting their growth performance. The intervention of probiotic fermentation in coconut water led to a reduction in the levels of immune indicators in the serum, promoting the recovery of intestinal inflammation.

Increased levels of inflammatory factors led to similar damage in the liver. This finding corroborates previous reports that DSS ingestion elevates serum D-lactic acid levels, contributing to intestinal barrier dysfunction [15]. This study further corroborates this observation.

The intervention with MS2c in the ileum tissue showed more pronounced effects on tissue recovery compared to the biochemical indicators. It rapidly facilitated the restoration and repair of intestinal epithelial cells, promoting the integrity of the intestinal structure and aiding in the recovery of damaged tissues. In contrast, the interventions in the Y301 and mixed fermentation groups did not show as significant effects as the MS2c group. Histopathological results from the model group provide strong evidence for the successful establishment of the acute enteritis model.

Based on both intestinal wall thickness and HE staining results, it can be concluded that the intestinal tissue in the model group exhibited extensive damage. Acute inflammatory factors activated inflammatory responses in epithelial cells, causing damage to the mucosa and leading to decreased intestinal wall thickness [17,28]. Probiotic intervention promoted the recovery of acute enteritis, thus protecting the intestinal tissue from inflammation-induced damage. However, the protective effect of MS2c-fermented coconut water on the intestinal wall was relatively weaker due to its high organic acid content, which resulted in a lower pH. Consequently, its impact on intestinal wall thickness was not as pronounced as that observed in the MY5 group.

As per literature reports, the balance of the intestinal microbiota plays a crucial role in the pathogenic mechanisms in animals [25,29,30,31]. The intestinal microbiota is closely associated with intestinal inflammation, and the metabolites of the microbiota can influence the levels of inflammatory factors, thereby affecting the interaction between the host and microorganisms. Disruption of this delicate balance can lead to the development of intestinal inflammation. In this study, through metagenomic analysis of cecal contents, the intervention results with two strains revealed that MS2c-fermented coconut water positively impacted acute enteritis recovery by modulating intestinal microbiota and metabolites. *L*. *plantarum* MS2c increased the abundance of beneficial bacteria, including *L. plantarum*, the Christensenellaceae family, Among them, the Christensenellaceae family is known for its negative correlation with inflammation, which possesses potential probiotic characteristics [32,33]. The Lachnospiraceae family promotes gut health by producing short-chain fatty acids through metabolism [34,35], while decreasing the abundance of the potentially pathogenic *Alistipes* genus and promoting intestinal stability [34,36]. Despite its reported protective effects in some diseases, *Alistipes* has been shown to exhibit pathogenicity in enteritis and intestinal cancer [37]. Moreover, it elevated the levels of metabolites, such as glycerol ester CDP-DG (a-25:0/a-21:0), gentamicin C, polymyxin B2, and biotin sulphone, contributing to acute enteritis recovery. Notably, gentamicin C and polymyxin B2 are commonly used antibiotics in early poultry farming for treating intestinal inflammation. Whether these compounds are indigenous metabolites of fermented coconut water or produced by the intestinal microbial community cannot be determined due to the lack of data on fermented coconut water metabolites. This highlights a key limitation of this study.

Notably, the control group (CK2) exhibited significantly higher levels of 5-methoxytryptophan, known for its anti-inflammatory properties. This finding suggests that 5-methoxytryptophan might play a role in the immune response of chicks [38]. This is possibly due to its role as a metabolite involved in signaling pathways, with levels increasing in the gut during severe inflammation. Apigenin, well-known for its antiviral properties, was also found in higher abundance in the CK2 group. This suggests that the CK2 model chicks might utilize immune metabolic pathways to suppress inflammation [39]. Additionally, the well-known anti-inflammatory and antiviral metabolite 15-epi-lipoxin A4 is more abundant in the control group, suggesting that healthy chick intestines are naturally enriched with these protective molecules to resist invasion by pathogens.

The MS2c intervention, which had a significant impact on glycerophospholipid metabolism [22,40], resulted in elevated levels of CDP-DG (a-25:0/a-21:0). This metabolic shift exerted diverse effects, regulating hepatic lipid metabolism, boosting immunity, and exhibiting antioxidant capabilities. Consequently, it led to reduced serum levels of pro-inflammatory cytokines, such as IFN-γ, GPT, and GOT. This intervention further contributed to diminishing the pro-inflammatory cytokines IL-6 and TNF-α, thereby mitigating intestinal inflammation and mitigating damage to intestinal epithelial cells.

Y301 has the potential to promote acute enteritis recovery by modulating the abundance of the Lachnospiraceae. This modulation indirectly reduces the abundance of the *Alistipes* genus, resulting in an increase in compounds with anti-inflammatory and inflammation-mitigating properties. These compounds encompass melatonin, deoxyflavonucleoside, lauroylglycine, guanidinopropanoic acid, and methylserine. Existing reports highlight the positive correlation of the *Alistipes* genus with intestinal inflammation, suggesting its potential pathogenic role. Moreover, studies by Xu, K., and K. indicated that melatonin promotes cell proliferation and development in chicks, contributing to the recovery of chick growth performance [41].

Furthermore, Y301 intervention resulted in a reduction in the levels of pro-inflammatory metabolites such as tetradecanoic acid. This decrease in pro-inflammatory metabolites consequently led to lower levels of TNF-α, IFN-γ, and D-lactic acid, key factors in intestinal inflammation. These changes also impacted the alanine, aspartate, and glutamate metabolic pathways, resulting in increased levels of methionine and melatonin. In turn, these compounds enhanced the gut’s antioxidant capacity, playing a role in preserving gut health.

## 5. Conclusions

This study demonstrated the positive effects of probiotics and fermented coconut water on acute enteritis recovery in Wenchang chick. Probiotic-fermented coconut water administration improved growth performance by increasing feed intake and body weight. *S. cerevisiae* Y301 modulated amino acid metabolism, leading to higher melatonin levels that promoted cell proliferation and development. Additionally, MS2c intervention altered glycerophospholipid metabolism, increasing CDP-DG levels and enhancing the intestinal microbiota by reducing potentially pathogenic *Alistipes* and enriching beneficial *L. plantarum*. This intervention also generated therapeutic metabolites like gentamicin C and polymyxin B2, contributing to the reduction of inflammatory factors. The combination of these strains provided additive therapeutic benefits for acute enteritis by promoting growth performance and improving inflammatory responses.

## Figures and Tables

**Table 1:** Experimental group design table.

Enteritis Intervention Experiment Conducted on 14-Day-Old Chicks	Treatment Method and Duration.	
	1–7 Days	8–21 Days
blank control group (CK1)	Normal free access to water.	Normal free access to water.
model group (CK2)	Free access to 1.5% DSS in drinking water.	2 mL of drinking water.
the fresh coconut water group (CK3)	Free access to 1.5% DSS in drinking water.	2 mL of fresh coconut water.
*L. plantarum* MS2c group (M2)	Free access to 1.5% DSS in drinking water.	2 mL of MS2c-fermented coconut water.
*S. cerevisiae* Y301 group(Y3)	Free access to 1.5% DSS in drinking water.	2 mL of Y301-fermented coconut water.
the mixed group (MY5)	Free access to 1.5% DSS in drinking water.	2 mL of coconut water was fermented with a composite strains.

**Table 2:** DAI index scoring standard.

Grade	Percentage of Weight Gain Loss	Fecal Viscosity	Fecal Potential Blood
0	0	Normal	Negative
1	1–5%	Soft feces	Baby lue
2	5–10%	Mucous feces	BlueB
3	10–20%	Liquid feces	Dark blue
4	>20%		Obvious bloody stool

## Data Availability

The data presented in this study are available on request from the corresponding author. The data are not publicly available due to privacy.
